# Shikonin inhibits migration and invasion of triple-negative breast cancer cells by suppressing epithelial-mesenchymal transition via miR-17-5p/PTEN/Akt pathway

**DOI:** 10.7150/jca.47553

**Published:** 2021-01-01

**Authors:** Chang Bao, Tao Liu, Lingbo Qian, Chi Xiao, Xinru Zhou, Heng Ai, Jue Wang, Weimin Fan, Jie Pan

**Affiliations:** 1 School of Basic Medical Sciences & Forensic Medicine, Hangzhou Medical College, No.481 Binwen Road, Hangzhou 310053, People's Republic of China.; 2 Program of Innovative Cancer Therapeutics, Division of Hepatobiliary and Pancreatic Surgery, Department of Surgery, First Affiliated Hospital, College of Medicine, Zhejiang University, Hangzhou 310003, People's Republic of China.; 3 Key Laboratory of Organ Transplantation, Hangzhou 310003, People's Republic of China.; 4 Key Laboratory of Combined Multi-organ Transplantation, Ministry of Public Health, Hangzhou310003, People's Republic of China.; 5 Department of Respiratory Medicine, Hospital of Traditional Chinese Medicine of Pingxiang city, No.10 Pingchuxi Road, Pingxiang 337000, People's Republic of China.; 6 Department of Pathology and Laboratory Medicine, Medical University of South Carolina, Charleston, SC 29425, USA.

**Keywords:** Shikonin, Triple-negative breast cancer, Epithelial-to-mesenchymal transition, miR-17-5p, PTEN

## Abstract

**Background:** Triple-negative breast cancer (TNBC) is a great threat to global women's health due to its high metastatic potential. Epithelial-to-mesenchymal transition (EMT) is considered as a key event in the process of metastasis. So the pharmacological targeting of EMT might be a promising strategy in improving the therapeutic efficacy of TNBC. Here, we investigated the effect of shikonin exerting on EMT and consequently the metastasis of TNBC cells and its underlying mechanism.

**Methods:** The invasive and migratory capacities of MDA-MB-231 and BT549 cells were tested using transwell invasion and wound healing assay. MiR-17-5p expression was examined by qRT-PCR. MiR-17-5p targeted genes were predicted with different bioinformatic algorithms from four databases (TargetScan, miRanda, PITA and picTar) and further screened by Kyoto Encyclopedia of Genes and Genomes (KEGG) pathway enrichment analysis. The differential expressions of predicted genes and their correlations with miR-17-5p were identified in breast cancer patients based on The Cancer Genome Atlas (TCGA) database. The interaction between phosphatase and tensin homolog deleted on chromosome ten (PTEN) and miR-17-5p was analyzed by luciferase reporter assay. The overexpression vector and small interfering RNA were constructed to investigate the role PTEN played in metastasis and EMT regulation. The expressions of EMT markers, protein kinase B (Akt) and phospho-Akt (p-Akt) were evaluated by western blot.

**Results:** Shikonin suppressed the migration and invasion of MDA-MB-231 and BT549 cells and meanwhile the corresponding alterations of EMT biomarkers were observed in shikonin treated MDA-MB-231 cells. Shikonin inhibited the expression of miR-17-5p, which was upregulated in breast cancer. The 3'-untranslated region (3'-UTR) of PTEN was found to be direct binding target of miR-17-5p by luciferase reporter assays. PTEN functioned as a suppressor both in the metastasis and EMT of TNBC cells. Moreover, Akt and p-Akt (Ser473) were involved in the process of inhibition in cancer cell migration, invasion and EMT by shikonin.

**Conclusions:** Shikonin inhibits migration and invasion of TNBC cells by suppressing EMT via miR-17-5p/PTEN/Akt pathway. This suggests shikonin as a promising therapeutic agent to counteract metastasis in the TNBC patients.

## Introduction

Breast cancer (BC) is the most common female malignant tumor and the leading cause of cancer related mortality among women worldwide^1^. In spite of the diverse therapeutic regimens available for BC, the incidence of treatment failure and cancer recurrence remains high. Approximately up to 90% of death of patients with BC is caused by invasion and metastasis^2^. Particularly, the morbidity of triple-negative breast cancer (TNBC) is higher than that of other BC subtypes primarily due to its high metastatic potential^3^, ^4^. TNBC, accounting for 10-20% of all breast cancers, is characterized by its lack of expression of the progesterone receptor (PR), estrogen receptor (ER) and human epidermal growth factor receptor 2 (HER2)^5^. The lack of therapeutic targets and proneness to metastasis and chemoresistance make it an aggressive breast cancer subtype with fewer effective treatment options and subsequently poor prognosis^6^. The prevalence of TNBC in younger and obese women is higher than hormone receptor-positive breast cancers and the relapse/recurrence and death rates of TNBC are high among patients 3-5 years after surgery^7^. Thus, TNBC is a great threat to global women's health. It is imperative to elucidate the mechanisms underlying TNBC metastasis.

Metastasis is a multistep process, in which epithelial-to-mesenchymal transition (EMT) is considered as a key event. EMT is defined as the loss of epithelial phenotypes and the gain of mesenchymal characteristics^8^. These changes endow cancer cells with higher metastatic capacity and contribute to abnormal cellular growth, differentiation and drug resistance^9^. Some signaling pathways are previously reported to be involved in EMT, such as transforming growth factor-β (TGF-β), cadherin, Notch and Wnt/β-catenin^2^, ^10^, ^11^. In breast cancer, forthcoming evidences suggest that EMT facilitates the metastatic dissemination of cancer cells *in vitro*, *in vivo* and in the clinical setting^12^, ^13^. So the pharmacological targeting of EMT might be a promising strategy in improving the therapeutic efficacy of TNBC.

To this end, many researchers have aimed to restrain EMT of cancer cells by certain agents with low toxicity and high efficiency. Shikonin (SHK), a naturally occurring compound, is extracted from the roots of Purple Cromwell, a kind of traditional Chinese herb used for a long time^14^. SHK has been found to exert anti-cancer effects by inducing apoptosis and inhibiting proliferation, metastasis and drug resistance of cancer cells in various malignancies including breast cancer^15^-^17^. Of particular interest, it is reported that the EMT process might be a target of SHK in reducing metastasis in some cancers, such as cervical and lung cancer^18^, ^19^. However, the effect of SHK on TNBC cells and its underlying mechanism is not fully understood. Additionally, it is known that the development of EMT is regulated by some cancer-relevant microRNAs (miRNAs), such as miR-9, miR-181a, miR-221, miR-155, miR-10b^20^, ^21^. However, to the best of our knowledge, few reports focus on the miRNA involvement in the EMT regulation of SHK.

In the current study, we hypothesized that SHK could reduce migration and invasion of human TNBC cells via the suppression of EMT by inhibiting miRNA expression and provided experimental validation for it. Herein, it is confirmed that SHK effectively inhibits the migration and invasion of human TNBC cell line by suppressing EMT. In examining the mechanism whereby SHK functions in BC cell metastasis, a novel miR-17-5p/PTEN/Akt pathway was identified in the regulation of EMT.

## Materials and Methods

### Cell culture and reagents

MDA-MB-231 and BT549 cell lines purchased from the Cell Bank of the Chinese Academy of Sciences (Shanghai, China) were maintained in Dulbecco's modified Eagle's medium (DMEM, Gibco, Grand Island, NY, USA) and Roswell Park Memorial Institute (RPMI) 1640 medium (Gibco, USA) respectively supplemented with 10% fetal bovine serum (Gibco, USA) and 1% penicillin/streptomycin antibiotics. Experiments were initiated when the cells exhibited logarithmic growth. SHK was purchased from Selleck Chemicals (Houston, TX, USA) and stored at -20°C. It was dissolved in dimethyl sulfoxide (DMSO) to a 50 mM stock. The storage solution was diluted to required concentrations just before each experiment. And the cells were treated with various concentrations of SHK for 24 h.

### Cell viability assay

Cell viability was examined by the microculture tetrazolium (MTT) assay. Cells were evenly seeded into 96-well plates with 2 × 10^3^ cells per well and incubated for one night. Then, each column was treated with different concentrations of SHK or control medium for 24 h. 4 h prior to the endpoint, the MTT solution was added into each well. After additional 4 h incubation at 37°C in the dark, the absorbances of the individual wells were measured at 570 nm wavelength with a microplate reader (Bio-Rad, Sunnyvale, CA, USA).

### Transwell invasion assay

The invasion of TNBC cells was evaluated by the matrigel-coated transwell chamber (Corning, MA, USA). Briefly, cells were seeded in the upper chambers at a density of 2×10^5^ cells/well and cultured in a serum-free medium with or without series concentrations of SHK for 24 h. The bottom chambers were filled with complete DMEM or RPMI medium. After incubation for 24 h, the invaded cells on the lower surfaces of the membranes were fixed with 100% methanol for 10 min at room temperature and then stained with 0.1% crystal violet for 15 min. The invaded cells were counted using a light microscope (Olympus, Japan).

### Wound healing assay

MDA-MB-231 or BT549 cells were seeded into the six-well plates with a density of 2×10^4^ cells per well in complete DMEM or RPMI medium respectively. When the cells grew to 100% confluence, the cell monolayer was scrapped with a sterile micropipette tip to create wounds. The wells were washed with phosphate buffer saline (PBS) and the remaining cells continued to be cultured in mediums with or without series concentrations of SHK for 24 h. The scratch was observed and captured at the same location with a light microscope at 0 h and 24 h after scrap. The gap distance of each wound was measured.

### Plasmids

For gene knockdown experiments, PTEN siRNA (sense: CGCCAAAUUUAAUUGCAGATT; anti-sense: UCUGCAAUUAAAUUUGGCGTT) and negative control siRNA were obtained from GenePharma (Shanghai, China). For gene overexpression vector construction, the open reading frames and downstream 3'-UTR of PTEN were cloned into the pcDNA3.1 vector (Invitrogen, Carlsbad, CA, USA) between the HindIII and EcoRI sites and driven by the cytomegalovirus promoter. For the luciferase reporter assay, the 3'-UTR fragment of PTEN was amplified and cloned into the XhoI and NotI sites downstream of the SV40 promoterdriven Renilla luciferase cassette in the psiCHECK-2 plasmid (Promega, Madison, WI, USA). A Fast Mutagenesis kit (Vazyme Biotech, Nanjing, China) was used to mutate the miR-17-5p binding sites in the PTEN 3'-UTR vectors according to the manufacturer's instructions.

### Transient transfection of miRNA

MDA-MB-231 cells were seeded in 6-cm cell culture dishes at 1.2×10^5^ cells/mL. The cells were transfected with a 50nM miR-17-5p mimic, miR-17-5p inhibitor or the corresponding scrambled negative control (NC) (Ribobio, Guangzhou, China) with lipofectamine 2000 (Invitrogen) per the manufacturer's instructions. After 24 h, the medium was replaced and the cells were prepared for subsequent experiments.

### Western blot assay

After pretreated with SHK or transfected with miRNA, siRNA or expression vectors, MDA-MB-231 cells were harvested and lysed in radioimmunoprecipitation assay (RIPA) buffer (Beyotime Institute of Biotechnology, China) to extract the total proteins. The protein content was detected by using the bicinchoninic acid (BCA) kit (Thermo Fisher Scientific, MA, USA). Equal amounts of denatured proteins (40μg) were electrophoresed on 10% polyacrylamide gel and subsequently transferred onto polyvinylidene fluoride membranes (Roche, Switzerland). After being blocked in Tris-Buffered Saline containing 0.1% Tween-20 (TBST) for 1 h, the membranes were probed with specific antibodies against PTEN (32072, Abcam, Cambridge, UK), N-cadherin (4061, Cell Signaling Technology, MA, USA), Vimentin (3390, Cell Signaling Technology), E-cadherin (5296, Cell Signaling Technology), Akt (9272, Cell Signaling Technology), p-Akt (4051, Cell Signaling Technology), GAPDH (A2228, Sigma-Aldrich) at 4℃ overnight respectively. After washing with TBST for three times, the membranes were incubated with an optimal dilution of the appropriate secondary antibodies conjugated with horseradish peroxidase (HRP) for 2 h at the room temperature. At last, the immunoreactive bands were detected with enhanced chemiluminescence reagents (Thermo Scientific, Darmstadt, Germany) by ChemiDoc Touch Imaging System (Bio-Rad, CA, USA) according to the manufacturer's instructions. Experiments were repeated independently at least three times.

### RNA extraction and quantitative reverse transcription-polymerase chain reaction (qRT-PCR)

Total RNA was extracted from BC cells with RNAiso Plus (TaKaRa, Kusatsu, Japan). The extracted RNA was reverse-transcribed into cDNA with a PrimeScriptTM RT reagent Kit (#RR037A, TaKaRa). Random hexamer primers and specific primers (Ribobio) were used for RNA and miRNA reverse-transcriptase PCR (RT-PCR) respectively.

qRT-PCR was performed to examine the expression of PTEN and miR-17-5p. Hypoxanthine phosphoribosyltransferase 1 (HPRT1) and U6 were used as internal control for PTEN and miR-17-5p expression, respectively. The specific primer sequences were as following: PTEN: 5'-CATGTTGCAGCAATTCACTG-3' (forward), 5'-CTTGTGAAACAACAGTGCCA-3' (reverse); HPRT1: 5'-TGACACTGGCAAAACAATGCA-3' (forward), 5'-GGTCCTTTTCACCAGCAAGCT-3' (reverse); U6: 5'-CTCGCTTCGGCAGCACATA-3' (forward), 5'-AACGCTTCACGAATTTGCG-3' (reverse). Mature miRNA expression was quantified by qRT-PCR with specific BulgeLoop miRNA qRT-PCR primers (Ribobio). qRT-PCR was performed in a LightCycler 480II system (Roche Diagnostics, Basel, Switzerland) with a SYBR Premix EX Tag kit (#RR420A, TaKaRa). Relative expression of RNA and miRNA were calculated as 2^-ΔCt^ after normalization with the reference control.

### Luciferase reporter assay

For reporter assay, MDA-MB-231 cells were transiently co-transfected with a 50nM miR-17-5p mimics or negative control-miR and 50ng of PTEN 3'-UTR wild-type or mutant psiCHECK-2 reporter vectors (Promega). Firefly luciferase activities were measured using the Dual Luciferase Reporter Assay System Kit (Promega) as described in the manufacturer's protocol 48 hours after transfection. Luminometry readings were obtained with a Varioskan Flash Spectral Scanning Multimode Reader (Thermo Scientific, Waltham, MA, USA). Firefly luciferase activity was normalized to constitutive Renilla luciferase. Each transfectant was assayed in triplicate.

### Bioinformatic analysis

MiR-17-5p targeted genes were predicted with different bioinformatic algorithms from various databases, including TargetScan (http://www.targetscan.org/), miRanda (http://www.microrna.org/microrna/home.do), PITA (http://genie.weizmann.ac.il/pubs/mir07/mir07_data.html) and picTar (https://pictar.mdc-berlin.de/). The visualization of Encyclopedia of Genes and Genomes (KEGG) pathway enrichment analysis for the common predicted genes used R package clusterProfiler^22^ from the bioconductor project. Adjust *p* value < 0.05 was considered as statistically significant.

The miRNA and mRNA expression data of breast cancer samples measured by Illumina-Hiseq were retrieved from The Cancer Genome Atlas (TCGA) database (https://cancergenome.nih.gov/), including 1085 breast cancer samples and 291 normal samples. Data were sorted and normalized. MiRNA and mRNA differential expression and correlation analyses were conducted by the R package edgeR 23.

### Statistical analysis

All data were presented as mean ± SEM from at least three independent experiments. Statistical analyses were performed with GraphPad Prism Ver. 5.01 (San Diego, CA, USA). Statistical significance was assessed with two-tailed Student's t-test or one-way ANOVA analysis. Pearson's correlation was used to evaluate the correlation between two variants. Statistical significance was set at *p* < 0.05 and displayed as *** for* p*<0.001; ** for *p*<0.01 and * for* p*<0.05.

## Results

### SHK inhibits the migration, invasion and EMT of human TNBC cells

**Figure 1:**
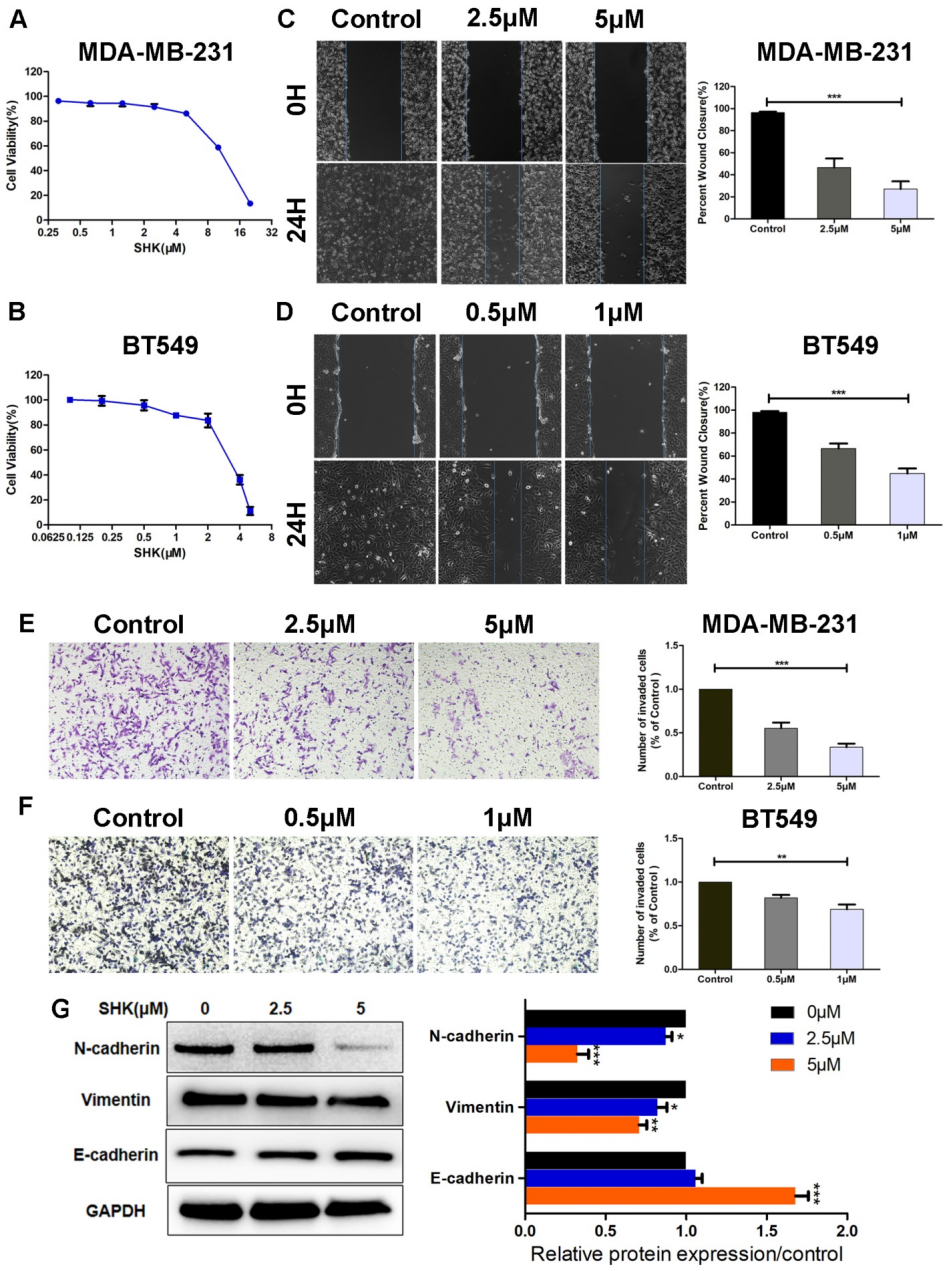
SHK inhibits the migration, invasion and EMT of TNBC cells. (A, B) Breast cancer cells were treated with various concentrations of SHK or control medium for 24 h. The effects of SHK on cell viability of MDA-MB-231 (A) and BT549 (B) were determined with MTT assays. (C, D) MDA-MB-231 and BT549 cells were treated with SHK (MDA-MB-231 at 2.5 and 5μM, BT549 at 0.5 and 1μM) or the control for 24 h. The migration of MDA-MB-231 (C) and BT549 (D) cells were detected by wound-healing assay (left) and the wound closures were quantified (right). (E, F) MDA-MB-231 and BT549 cells were treated as in (C, D). The invasion of MDA-MB-231 (E) and BT549 (F) cells were detected by transwell invasion assay (left) and the invaded cells were quantified (right). (G) The EMT markers (N-cadherin, Vimentin and E-cadherin) protein levels were analyzed by Western blot (left) and the blots were further quantified with ImageJ (right). GAPDH is shown as a loading control. Error bars represent the SEM obtained from at least three independent experiments. *p<0.05, **p<0.01, ***p<0.001.

First of all, the effects of SHK on BC cell viability were determined with MTT assays. A 24-h cell viability assay revealed that, the IC50 of SHK was 11.3±1.43μM in MDA-MB-231 cells and 3.2±1.07μM in BT549 cells (Fig. 1A, B). Their viabilities differed a lot at the same concentration of SHK. To avoid the direct toxicity of SHK to the target cells, different concentrations were adopted in the following study. As shown in Figure 1A and 1B, the viability of MDA-MB-231 cells was 91.5±4.6% at 2.5μM SHK and 86.3±3.1% at 5μM. And in BT549 cells, the cell viability was 95.8±4.0% at 0.5μM and 87.8±2.1% at 1μM (Fig. 1A, B). Thus, at the concentrations used in this research, the direct toxicity of SHK to the target cells was limited.

To investigate the effects of SHK on cell migration and invasion, MDA-MB-231 cells were exposed to 2.5μM or 5μM SHK and BT549 cells to 0.5μM or 1μM SHK for 24 h. The capacities of migration and invasion were detected by wound‑healing assay and transwell invasion assay respectively. As shown in Figure 1C and 1D, the 24 h wound-healing assay revealed that the migratory capacity was decreased significantly with the treatment of SHK compared with that of cells in the control (Fig. 1C, D). Similarly, SHK treatment suppressed invasion of breast cancer cells in a dose-dependent manner (Fig. 1E, F). These data indicate that SHK decreases the migratory and invasive capacity of breast cancer cells.

As EMT was always considered as a pivotal event that occurs during cancer metastasis, the expression of EMT markers were further explored by western blot. The results showed that the protein expressions of mesenchymal markers N-cadherin and vimentin were downregulated in the SHK-treated group, whereas that of the epithelial marker E-cadherin was upregulated with the treatment of SHK (Fig. 1G).

### SHK reduces the expression of miR-17-5p that promotes TNBC cell migration, invasion and EMT

**Figure 2:**
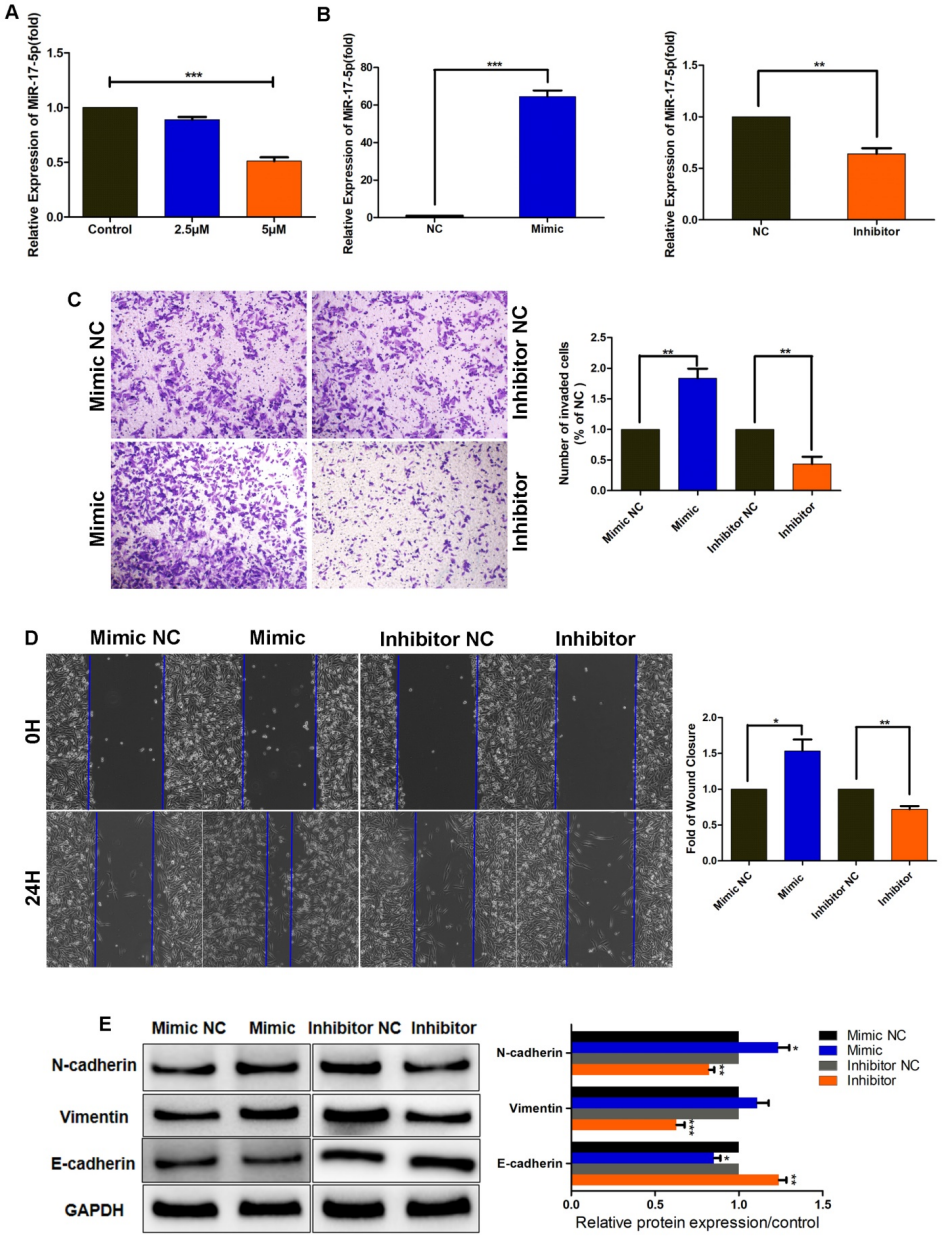
MiR-17-5p promotes TNBC cell migration, invasion and EMT. (A) MDA-MB-231 cells were pretreated with different concentrations of SHK (2.5 and 5μM) or the control for 24 h. The level of miR-17-5p was determined by qRT-PCR, with U6 as a reference. (B-E) MDA-MB-231 cells were transfected with a 50nM miR-17-5p inhibitor, mimic or the corresponding negative control (NC) for 24 h. (B) The transfection efficiency was assessed by qRT-PCR, with U6 as a reference. (C) The cell invasion was detected by transwell invasion assay (left) and the invaded cells were quantified (right). (D) The cell migration was detected by wound-healing assay (left) and the wound closures were quantified (right). (E) The protein levels of N-cadherin, Vimentin and E-cadherin were analyzed by Western blot (left) and the blots were quantified with ImageJ (right). GAPDH is shown as a loading control. Data represent the mean ± SEM of three independent experiments. *p<0.05, **p<0.01, ***p<0.001

To further elucidate the function of SHK in inhibiting migration and invasion, we examined the expression of miR-17-5p in MDA-MB-231 cells pretreated with SHK. When BC cells were treated with SHK (2.5μM or 5μM) for 24 h, the level of miR-17-5p declined dose-dependently (Fig. 2A), which confirmed the inhibitory regulation of SHK on miR-17-5p expression.

To investigate the effect of miR-17-5p on cell migration and invasion; we transiently transfected MDA-MB-231 cells with miR-17-5p mimics, miR-17-5p inhibitors, or the corresponding negative control (NC), and assessed the efficiency of transfection by qRT-PCR. The results revealed that the level of miR-17-5p was increased by miR-17-5p mimics but decreased by miR-17-5p inhibitors (Fig. 2B). Then, the invasive and migratory abilities of the transfected cells were tested. Compared with the control group, the number of cells traversing the matrigel was significantly increased in miR-17-5p mimic-transfected group, and the opposite results were observed in miR-17-5p inhibitor-transfected group (Fig. 2C). The wound-healing ability was enhanced in cells transfected with miR-17-5p mimics, but was attenuated in those transfected with miR-17-5p inhibitors (Fig. 2D). Next, the regulation of miR-17-5p on EMT markers was detected by western blot. Accordingly, the expressions of N-cadherin and vimentin were upregulated in MDA-MB-231 cells transfected with miR-17-5p mimics, while downregulated in miR-17-5p inhibitors transfected group. And the level of E-cadherin was decreased by miR-17-5p mimics and increased by miR-17-5p inhibitors, which exhibited an opposite expression pattern with N-cadherin and vimentin (Fig. 2E). Thus, these data demonstrate that miR-17-5p may promote the migration, invasion and EMT of breast cancer cells, and SHK reduces these processes by downregulating miR-17-5p.

### PTEN is screened to be the target of miR-17-5p using bioinformatic analyses

**Figure 3:**
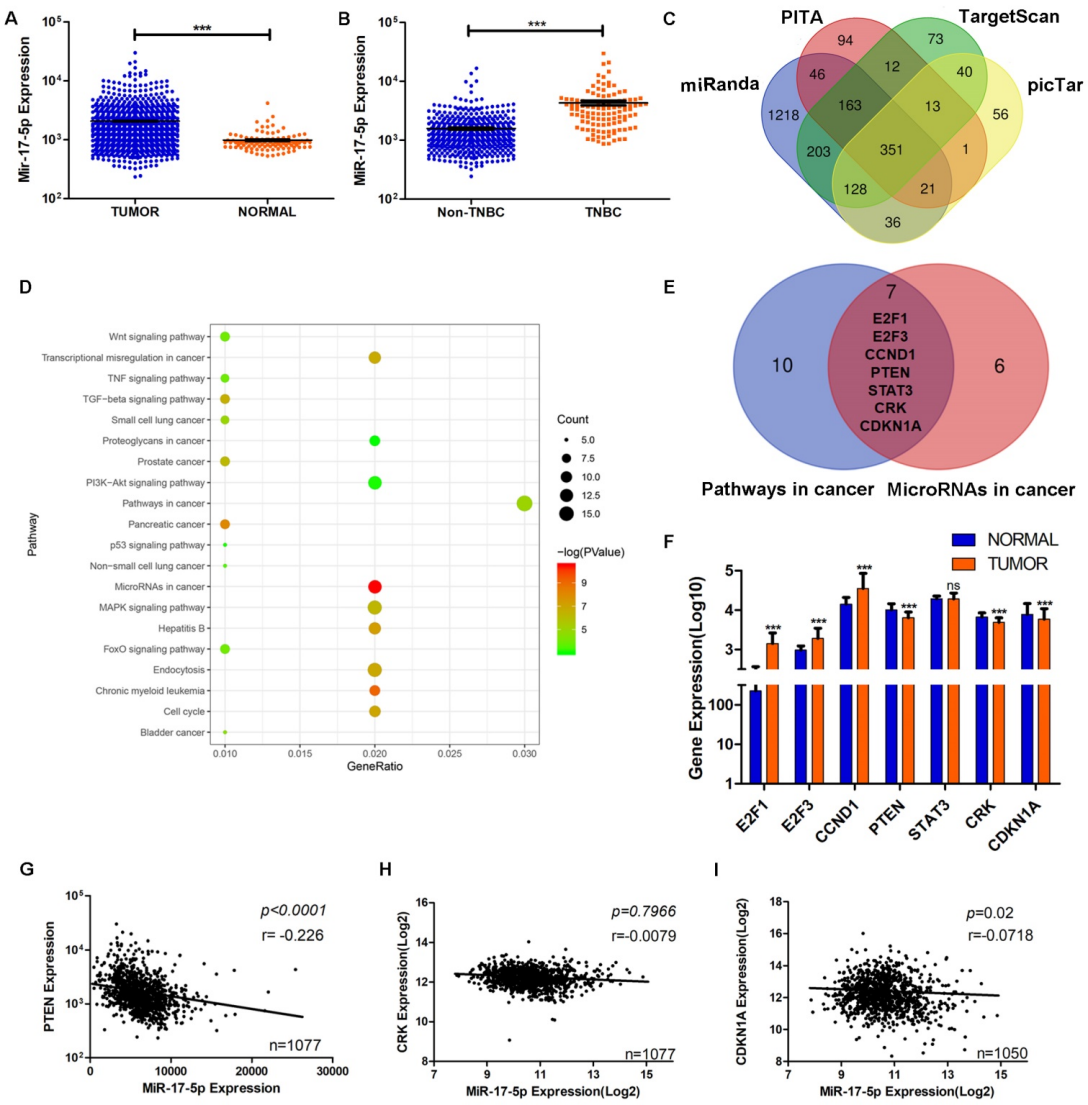
The screening of target gene of miR-17-5p in breast cancer cell migration and invasion. (A) The expression of miR-17-5p was analyzed in 1085 breast cancer and 291 normal breast tissue samples from TCGA. (B) The expression of miR-17-5p was analyzed in 494 non-TNBC and 113 TNBC tissue samples from TCGA. (C) 351 genes were overlapped in the four groups of predicted target genes from TargetScan, PITA, miRanda and picTar. (D) The enrichment analysis of KEGG pathways was performed in the 351 genes. (E) Seven genes were overlapped between the two relevant pathways - microRNAs in cancer and pathways in cancer. (F) The expressions of the seven genes were analyzed in BC (n=1085) and normal (n=291) cohorts from TCGA. (G-I) The correlation between miR-17-5p and PTEN (G), CRK (H) or CDKN1A (I) in BC cohort from TCGA. ***p <0.001.

Data from TCGA showed the level of miR-17-5p was significantly higher in BC group (n=1085) than the normal group (n=291) (Fig. 3A). Compared with other subtypes of BC, miR-17-5p expression was higher in TNBC (Fig. 3B). And we had already reported that the cancer recurrence rate was higher and the overall survival probability was poorer in patients with higher levels of miR-17-5p than in those with lower levels 24. Although miR-17-5p had been reported to contribute to tumorigenesis and progression in various cancers, the underlying mechanism of its effects on breast cancer cell migration and invasion remains to be elucidated. To this end, by analyzing the data from four public databases (TargetScan, PITA, miRanda and picTar), we acquired 351 predicted target genes of miR-17-5p in common (Fig. 3C). In order to better understand the biological features and significances of the 351 genes, the enrichment analysis of KEGG pathways was performed and indicated that the genes were enriched in multiple cancer-related pathways (Fig. 3D). Venn diagram showed 7 genes were overlapped between the two relevant pathways - microRNAs in cancer and pathways in cancer (Fig. 3E). Then, the differential expressions of the 7 genes were analyzed in BC and normal cohorts from TCGA. It demonstrated that PTEN, CRK and CDKN1A were downregulated significantly in BC compared with the normal group (Fig. 3F). According to the characteristics of microRNA in regulating its target genes, PTEN, CRK and CDKN1A were chosen as the potential target genes, as their differential expression patterns were conformed to the effect of miR-17-5p. Next, the correlations of miR-17-5p with the potential target genes were investigated in the BC samples from TCGA's cohort (Fig. 3G-I). As shown in Fig. 3G, statistical analysis revealed a significant inverse correlation between the expression levels of endogenous miR-17-5p and PTEN, which pointed toward the probable involvement of miR-17-5p in PTEN regulation.

### MiR-17-5p downregulates PTEN by directly binding to its 3'-UTR

**Figure 4:**
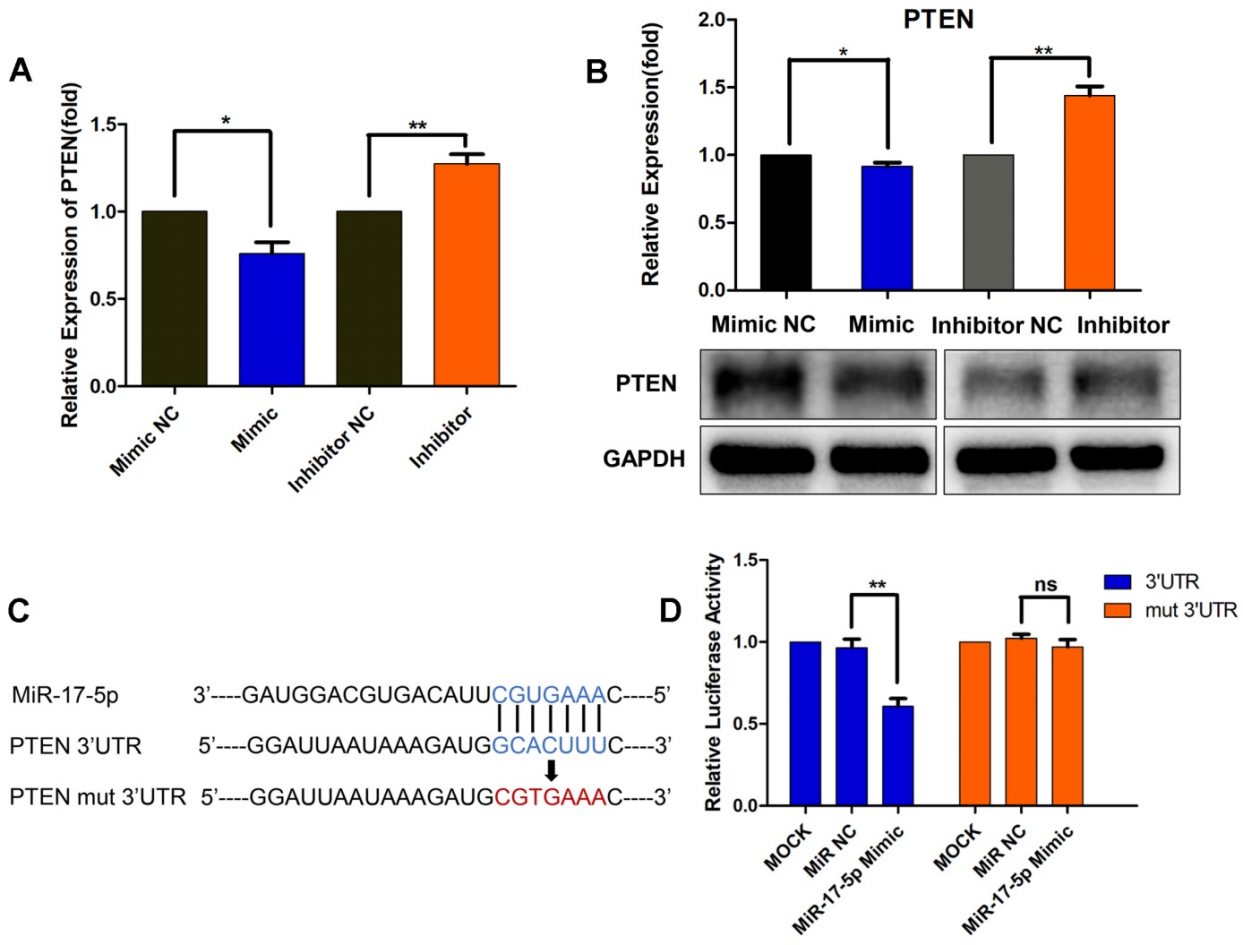
MiR-17-5p downregulates PTEN by directly binding to its 3'-UTR. (A-B) MDA-MB-231 cells were transfected with a 50nM miR-17-5p inhibitor, mimic or the corresponding negative control (NC) for 24 h. The PTEN mRNA (A) and protein (B) levels were detected by qRT-PCR and Western blot respectively. (C) Schematic representation of predicted miR-17-5p binding sites in the 3'-UTR of PTEN and 3'-UTR mutated alignment. (D) Luciferase reporter assay of MDA-MB-231 cells co-transfected with a miR-17-5p mimic, NC or mock and either wild-type or mutated luciferase plasmid. Error bars represent the SEM obtained from three independent experiments. *p<0.05, **p<0.01.

As a result, we focused on PTEN, which was a well-known tumor suppressor. As presented in Fig. 4A and 4B, miR-17-5p mimic transfection resulted in a significant decrease in the mRNA and protein levels of PTEN, whereas the inhibitor-transfected group showed increased PTEN expression (Fig. 4A, B). These results further suggested the regulation of miR-17-5p on PTEN expression. Though miR-17-5p was revealed to have a putative binding site in the 3'-UTR of PTEN, further luciferase reporter assays were required to validate whether PTEN was a functional target of miR-17-5p in MDA-MB-231 cells. Wild-type 3'-UTR of PTEN and mutated sequence (mut PTEN 3'-UTR) were individually cloned into reporter vectors downstream of the Renilla luciferase gene (Fig. 4C). MDA-MB-231 cells were transfected with a reporter vector only, or co-transfected with the reporter vector and either 50nM miR-17-5p or NC. The luciferase activity of the reporters with wild-type PTEN 3'-UTR was significantly suppressed by miR-17-5p mimic transfection, while the activity of the reporters with mutant PTEN 3'-UTR did not change significantly (Fig. 4D). These results demonstrate that miR-17-5p downregulates PTEN by directly binding to its 3'-UTR.

### Downregulation of PTEN increases the migration, invasion and EMT of breast cancer cells

**Figure 5:**
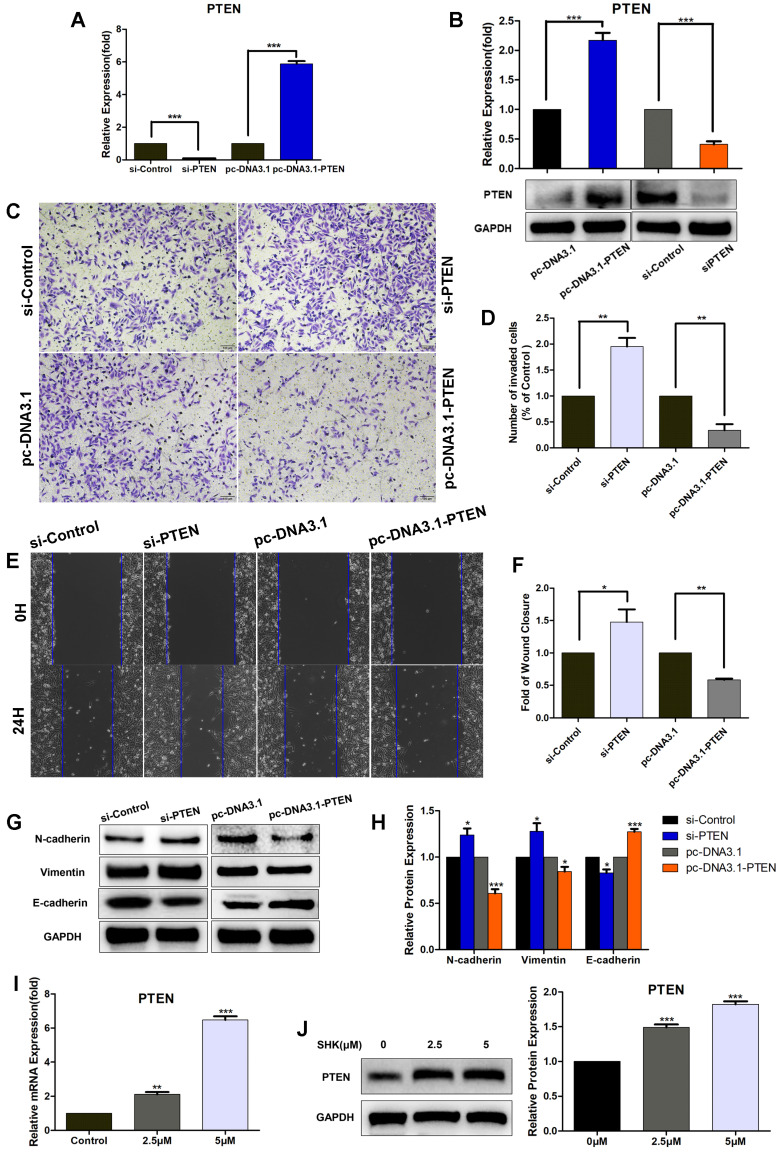
Downregulation of PTEN increases the migration, invasion and EMT of TNBC cells and SHK upregulates PTEN expression. (A-H) MDA-MB-231 cells were transfected with control siRNA (si-control) or PTEN siRNA (si-PTEN) and with PTEN overexpression vector (pc-DNA3.1-PTEN) or control vector (pc-DNA3.1). (A) The expression of PTEN mRNA was determined by qRT-PCR, with HPRT1 as a reference. (B) The expression of PTEN protein was determined by western blot and the blots were quantified by using ImageJ. (C) The cell invasion was detected by transwell invasion assay. (D) Quantification of the invaded cells. (E) The cell migration was detected by wound-healing assay. (F) Quantification of the wound closures. (G, H) The expressions of N-cadherin, Vimentin and E-cadherin were analyzed by Western blot (G) and further quantified with ImageJ (H). GAPDH is shown as a loading control. (I, J) MDA-MB-231 cells were pretreated with different concentrations of SHK (2.5 and 5μM) or the control for 24 h. PTEN expression was evaluated at mRNA (I) and protein (J) levels. Data represent the mean ± SEM of three independent experiments. *p<0.05, **p<0.01, ***p<0.001

In order to evaluate the role of PTEN in breast cancer cell migration and invasion, we transfected MDA-MB-231cells with PTEN siRNA (si-PTEN) or overexpression vectors (pc-DNA3.1-PTEN) to knock down or overexpress PTEN. In MDA-MB-231 cells, the mRNA and protein expression of PTEN was significantly lower in the PTEN siRNA transfected group and higher in PTEN overexpression vectors transfected group, compared with their corresponding control groups, which confirmed the efficiency of knockdown or overexpression (Fig. 5A, B). The wound healing and transwell assays showed the migration and invasion abilities were upregulated in cells transfected with si-PTEN and on the other hand, both were downregulated in cells transfected with pc-DNA3.1-PTEN (Fig.5C-F). Moreover, the expressions of N-cadherin and vimentin were inhibited in PTEN-overexpressing group and increased in PTEN-knockdown group. But contrary to that, an opposite pattern was observed in E-cadherin expression (Fig.5G, H). Altogether, these results show that PTEN may play an important role in suppressing MDA-MB-231 cell migration and invasion via inhibiting EMT.

Furthermore, we investigated whether SHK could regulate the expression of PTEN directly. When MDA-MB-231 cells were treated with SHK at different concentrations for 24 h, SHK was found to upregulate PTEN expression in both mRNA and protein levels dose-dependently (Fig. 5I, J).

### Akt is involved in the inhibition of SHK on cancer cell migration, invasion and EMT via miR-17-5p/PTEN pathway

**Figure 6:**
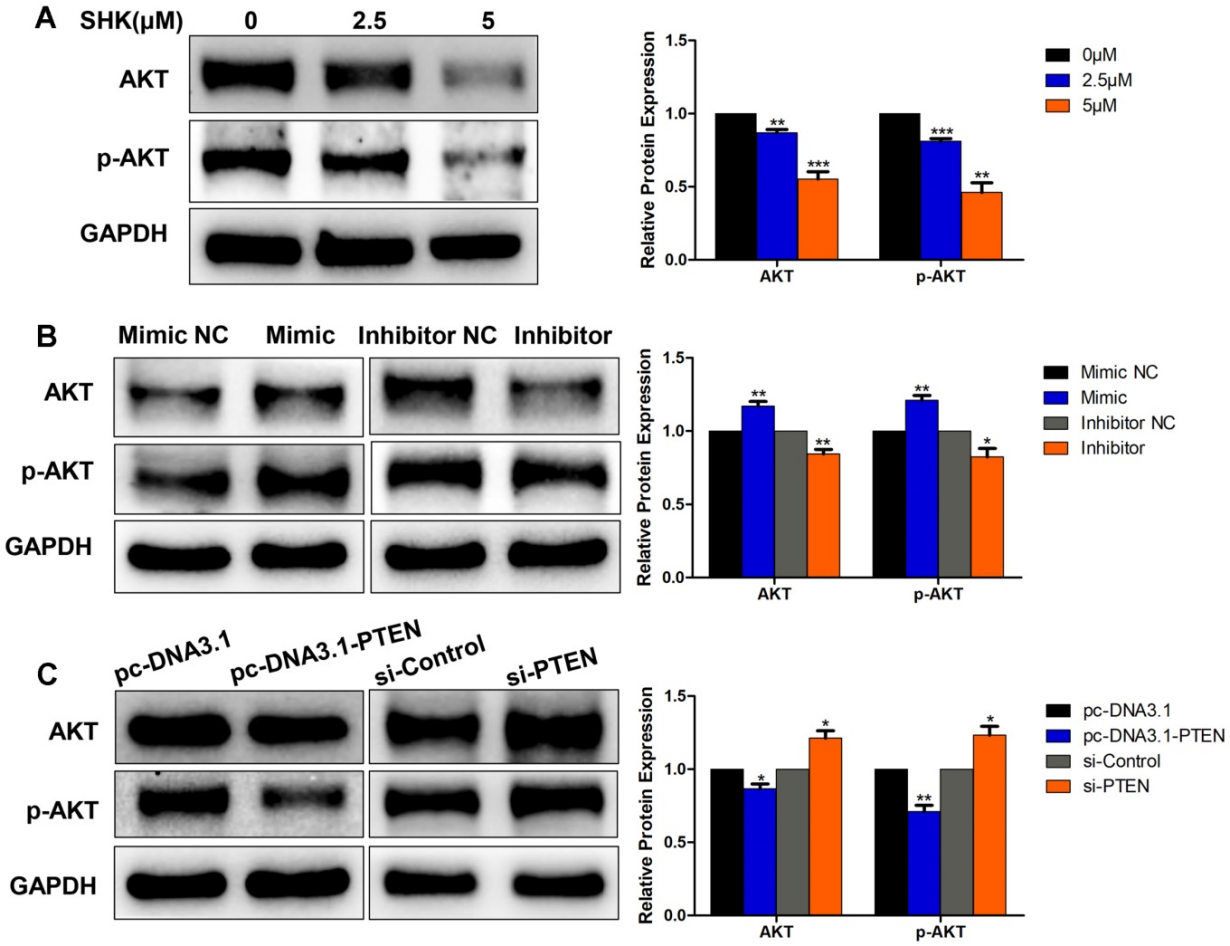
Akt involvement in the process of EMT. (A) The expressions of Akt and p-Akt were detected by western blot in MDA-MB-231 cells treated with SHK for 24 h. (B)The levels of Akt and p-Akt were determined in MDA-MB-231 cells transfected with miR-17-5p mimic, inhibitor and NC. (C) The expressions of Akt and p-Akt were detected in MDA-MB-231 cells transfected with si-control or si-PTEN and with pc-DNA3.1 or pc-DNA3.1-PTEN. All of the blots were quantified by using ImageJ. Data represent the mean ± SEM of three independent experiments. *p<0.05, **p<0.01, ***p<0.001

The phosphatidylinositol 3-kinase (PI3K) / protein kinase B (Akt) pathway is crucial in the regulation of cancer development and the activation of Akt subsequently leads to a number of potential downstream effects including cell metastasis. As PTEN acted as a major brake of this pathway, we hypothesized that Akt might function in the process of migration and invasion inhibition by SHK. To test this hypothesis, we investigated the effects of SHK, miR-17-5p and PTEN on Akt expression in MDA-MB-231 cells. Western blot showed Akt and phospho-Akt (p-Akt, Ser473) expression levels were downregulated in parallel with the increase in the concentration of SHK (Fig. 6A). Compared with the negative control groups, levels of Akt and p-Akt were significantly higher in the miR-17-5p mimic transfected group and lower in the miR-17-5p inhibitor transfected group (Fig. 6B). Overexpression of PTEN reduced the expression of Akt and p-Akt. On the other hand, knockdown of PTEN increased Akt and p-Akt expression (Fig. 6C). These observations suggest that Akt might play a role in inhibiting the migration and invasion of MDA-MB-231 cells by SHK to some extent.

## Discussion

Breast cancer is a heterogeneous disease with distinct biological properties, specific morphological patterns and diverse clinical behaviors among different subtypes. TNBC is the most aggressive subtype with early relapse, poor overall survival and, most importantly, frequent distant metastasis^25^. EMT is critical in the initiation of metastasis in TNBC because it enables BC cells to detach from the primary tumor and eventually to colonize distant organs^26^. Once the EMT process was inhibited, the metastasis of TNBC was suppressed or even reversed at the same time^27^, ^28^. So, it seems to be feasible to inhibit cancer cell metastasis by targeting EMT.

SHK is a natural bioactive compound with multiple health-promoting effects, mainly wound-healing, anti-inflammation, anti-infection and anti-cancer^29^, ^30^. Administration of SHK and its derivatives at a high dose is verified to be safe and well-tolerated in animal models^31^, ^32^. Thus far, several studies reported that SHK could inhibit tumorigenesis and progression of breast cancer. It was confirmed to reduce breast cancer stem cell load and tumorigenic potential by inhibiting STAT3, FAK and Src mediated pathways and estrogen signaling^33^-^35^. SHK induced cell cycle arrest and p38-dependent apoptosis in breast cancer^36^, ^37^. It also enhanced sensitivity of breast cancer cells to endocrine therapy and paclitaxel 38, 39. In addition, SHK was found to inhibit metastasis of breast cancer cell via GSK-3β-regulated suppression of β-catenin signaling and matrix metallopeptidase 9 (MMP-9) inhibition^40^, ^41^. Accordingly, SHK was shown to inhibit the migration and invasion of TNBC cell lines effectively in the present study, indicating that the use of SHK might provide an effective way to reduce metastasis of TNBC. In consideration of the important role EMT played in cancer metastasis, we supposed whether the anti-metastasis effect of SHK was mediated by regulating the process of EMT. Therefore, in this study, we confirmed that TNBC cells acquired the epithelial phenotypes and lost the mesenchymal feature with the treatment of SHK, which was in accordance with the previous studies in cervical and lung cancers^18^, ^19^.

Furthermore, the underlying mechanism whereby SHK inhibited EMT of TNBC cells was explored. MiRNAs are a group of endogenous RNA molecules participating in a variety of biological processes, such as cell cycle control, proliferation, apoptosis and migration^42^. Thus far, miR-17-5p has been reported to dysregulate in various malignancies and promote invasion and migration of cancer cells^43^, ^44^ . Data from TCGA showed that miR-17-5p was higher in breast cancer tissues than in normal breast tissues and was also higher in TNBC than non-TNBC. High expression of miR-17-5p might predict a poor prognosis in breast cancer patients 24. Herein, SHK was proven to downregulate the expression of miR-17-5p effectively with a dose-dependent manner in MDA-MB-231, indicating its inhibition of metastasis in TNBC might be mediated by miR-17-5p. We discovered that high level of miR-17-5p contributed to the EMT process and the invasion and migration of TNBC cells.

MiRNAs regulate the expression of target genes by binding sequence-specifically to the 3'-UTR of their mRNAs. By bioinformatic analyses, PTEN was predicted to be the target gene of miR-17-5p in modulating BC cell metastasis. And the expression of PTEN was correlated inversely with that of miR-17-5p in 1077 BC cancer samples from TCGA. The expression of PTEN was upregulated in response to SHK treatment, an effect that associated inversely with miR-17-5p expression. Then, miR-17-5p was verified to decrease PTEN expression via binding to its 3'-UTR with the dual luciferase reporter assay, which confirmed the direct targeting relationship between miR-17-5p and PTEN.

PTEN is a well-known tumor suppressor gene encoding a multifunctional protein that affects various biological activities including metastasis, which is always down modulated in human cancers^45^. Accordingly, it is found that PTEN expression is lower in BC cancer samples than in normal breast tissues. SHK could elevate the mRNA and protein level of PTEN dose-dependently in BC cells. The capacities of migration and invasion showed inverse correlations with the expression of PTEN in TNBC cells. PTEN downregulation or loss associates with the acquisition of EMT traits^46^. We discovered that the activity of EMT was increased or decreased in PTEN knocked-down or over-expressed BC cells respectively. Akt is key member of the PI3K/Akt axis, which is a major signaling pathway antagonized by PTEN 47. Akt activation via phosphorylation (p-Akt) controls metastasis in a diversity of tumors^48^, ^49^.The downregulation of PTEN in BC cells was in parallel with the upregulation of Akt and p-Akt (Ser473). Moreover, the levels of Akt and p-Akt (Ser473) were also decreased with SHK treatment and showed a positive correlation with miR-17-5p. These findings confirmed the involvement of Akt in the process of SHK regulating the invasion and migration via miR-17-5p/PTEN axis.

In conclusion, SHK suppressed EMT via miR-17-5p/PTEN/Akt pathway, thus consequently inhibiting migration and invasion of TNBC cells. Therefore, SHK could be a potential therapeutic agent to counteract metastasis in the TNBC patients.
